# Fungal elemental profiling unleashed through rapid laser-induced breakdown spectroscopy (LIBS)

**DOI:** 10.1128/msystems.00919-24

**Published:** 2024-08-27

**Authors:** Tomás A. Rush, Ann M. Wymore, Miguel Rodríguez, Jr, Sara Jawdy, Rytas J. Vilgalys, Madhavi Z. Martin, Hunter B. Andrews

**Affiliations:** 1 Biosciences Division, Oak Ridge National Laboratory, Oak Ridge, Tennessee, USA; 2 Biology Department, Duke University, Durham, North Carolina, USA; 3 Radioisotope Science and Technology Division, Oak Ridge National Laboratory, Oak Ridge, Tennessee, USA; University of California San Diego, La Jolla, California, USA

**Keywords:** fungal behavior, elemental profiling, ionomics, endophytes, poplar, LIBS

## Abstract

Elemental profiling of fungal species as a phenotyping tool is an understudied topic and is typically performed to examine plant tissue or non-biological materials. Traditional analytical techniques such as inductively coupled plasma–optical emission spectroscopy (ICP-OES) and inductively coupled plasma–mass spectrometry (ICP-MS) have been used to identify elemental profiles of fungi; however, these techniques can be cumbersome due to the difficulty of preparing samples. Additionally, the instruments used for these techniques can be expensive to procure and operate. Laser-induced breakdown spectroscopy (LIBS) is an alternative elemental analytical technique—one that is sensitive across the periodic table, easy to use on various sample types, and is cost-effective in both procurement and operation. LIBS has not been used on axenic filamentous fungal isolates grown in substrate media. In this work, as a proof of concept, we used LIBS on two genetically distinct fungal species grown on a nutrient-rich and nutrient-poor substrate media to determine whether robust elemental profiles can be detected and whether differences between the fungal isolates can be identified. Our results demonstrate a distinct correlation between fungal species and their elemental profile, regardless of the substrate media, as the same strains shared a similar uptake of carbon, zinc, phosphorus, manganese, and magnesium, which could play a vital role in their survival and propagation. Independently, each fungal species exhibited a unique elemental profile. This work demonstrates a unique and valuable approach to rapidly phenotype fungi through optical spectroscopy, and this approach can be critical in understanding these fungi's behavior and interactions with the environment.

**IMPORTANCE:**

Historically, ionomics, the elemental profiling of an organism or materials, has been used to understand the elemental composition in waste materials to identify and recycle heavy metals or rare earth elements, identify the soil composition in space exploration on the moon or Mars, or understand human disorders or disease. To our knowledge, ionomic profiling of microbes, particularly fungi, has not been investigated to answer applied and fundamental biological questions. The reason is that current ionomic analytical techniques can be laborious in sample preparation, fail to measure all potential elements accurately, are cost-prohibitive, or provide inconsistent results across replications. In our previous efforts, we explored whether laser-induced breakdown spectroscopy (LIBS) could be used in determining the elemental profiles of poplar tissue, which was successful. In this proof-of-concept endeavor, we undertook a transdisciplinary effort between applied and fundamental mycology and elemental analytical techniques to address the biological question of how LIBS can used for fungi grown axenically in a nutrient-rich and nutrient-poor environment.

## INTRODUCTION

Nutrient acquisition, delivery, and availability dictate microbes' phenotype, lifestyle, and survival. Among the best microbes with which to study the influence of substrate availability is fungi because they are heterotrophic, meaning they cannot produce their food from environmental nutrients; thus, these organisms derive their nutritional requirements from other complex organic plants or animals (1). Tapping into this biological process by manipulating the available substrate has led to discoveries in identifying how fungi interact with a host, environment, or biological products such as alternative sources of protein and natural products (2, 3). Nutrient acquisition and the diverse roles it plays in controlling fungal behavior and producing biological products, as well as the elements obtained and how they differ across fungal species, are underexplored topics in the literature. However, in the past few years, interest has grown in using different analytical techniques for elemental profiling of fungal species.

Previous studies investigating fungal elemental profiling (also known as *fungal ionomics*) employed traditional analytical techniques such as inductively coupled plasma–optical emission spectroscopy [ICP-OES, also known as *atomic emission spectroscopy* (AES)] and inductively coupled plasma–mass spectrometry (ICP-MS) (Table 1). Unless laser ablation sampling is used, both of these analytical techniques require complete digestion of the samples, which makes sample preparation laborious (4). However, recently, a few studies have used single cell-inductively coupled plasma-time-of-flight mass spectrometry (SC-ICP-TOF-MS) to analyze individual yeast cell ionomic profiles that do not require digestion (5, 6). Unfortunately, ICP-MS, ICP-TOF-MS, and coupled laser ablation introduction systems can be cost-prohibitive. Even in the case of ICP-OES, the operational costs (e.g., power, argon gas) can be expensive.

**TABLE 1:** Previous analytical tools used to determine fungal ionomic profiles^*a*^

Method	Fungi examined	Number of elements detected	Reference
HPLC with ICP-MS	*Aspergillus fumigatus, Candida albicans, Cryptococcus neoformans,* and *Saccharomyces cerevisiae*	10	7
ICP-OES	*Saccharomyces cerevisiae*	12	8
ICP-OES	*Agaricus bisporus*	6	9
ICP-OES	*Saccharomyces cerevisiae*	13	10
ICP-MS	*Cordyceps kyushuensis*	20	11
ICP-MS	*Hemileccinum impolitum, Butyriboletus roseoflavus,* and *Boletus umbriniporus*	19	12
ICP-MS	*Saccharomyces cerevisiae*	17	13
ICP-MS	*Schizophyllum commune*	4	14
ICP-MS	16 mushrooms	Various	15
ICP-MS and ICP-OES	*Perenniporia fraxinea*	11	16
ICP-OES	*Suillus variegatus*	18	17
ICP-OES	*Pleurotus* spp.	31	18
ICP-OES	30 edible mushrooms	15	19
LIBS	*Pleurotus opuntiae*	8	20
LIBS/SEM-EDS	*Candida* strains	4	21
LIBS/Raman/MS	Unknown	Various	22
SC-ICP-MS	*Saccharomyces cerevisiae*	Various	5
SC-ICP-TOF-MS	*Saccharomyces cerevisiae*	6	6

^*a*^ Note: HPLC = high-performance liquid chromatography.

We posit that laser-induced breakdown spectroscopy (LIBS) can be a robust optical method for elemental profiling because it requires little-to-no sample preparation, can detect both light and heavy elements, has low procurement and operational costs, and can rapidly produce large data sets with proper replications (23, 24). Recently, a study examined how *Pleurotus opuntiae* absorbs and removes lead contamination using LIBS (20). However, to our knowledge, no studies have investigated the sole use of LIBS in an untargeted fashion to determine fungal elemental profiles. To address this knowledge gap, we used LIBS to identify the fungal ionomic profiles of two genetically different fungal species grown on nutrient-rich and nutrient-poor media. These measurements were performed in a manner that can be scaled for high-throughput analysis in future studies.

Most fungal ionomics or elemental studies have focused on how edible fungi uptake trace elements, heavy metals, or exhibit tolerance to certain elements that could be harmful to humans if consumed (9, 11, 12, 14–20, 25). Other studies focused on the genetic model yeast organism, *Saccharomyces cerevisiae*, to correlate genes to the results of elemental profiling (13) in order to test single-cell analytic techniques (5, 6); to establish protocols to examine fungal ionomics (8); to test other detection methods such as hydride generation atomic absorption spectroscopy (HGAAS), which did not produce reliable results (26); or to test strain variation for the uptake and tolerance of various elements (10). Rarely have studies focused on how fungal species or strains' elemental profiles change when grown on different substrate media to answer biological questions about their growth and development. One of the few investigations that has focused on differences in the fungal ionomics profiles between strains was that of Eide et al. (10). They examined 4,385 *S*. *cerevisiae* mutant strains to understand differences in their ability to uptake nutrients while grown on different substrate media. This study used ICP-OES to detect 13 elements, and 4.83% of strains had an altered yeast ionome profiles when grown on a nutrient-rich medium (i.e., yeast peptone dextrose). In a separate study, LIBS was coupled with SEM-EDS in a clinical study to discriminate between *Candida* strains by distinguishing only carbon, hydrogen, nitrogen, and oxygen emission spectra (21). Lastly, LIBS was paired with Raman spectroscopy and mass spectrometry to understand elements obtained by fungi and how they degrade specialized coatings (i.e., epoxy primer-based coating used in marine and aviation applications) (22). Taken together, the literature's lack of attention on the sole use of LIBS to determine the elemental profile of fungi and the significant variation in nutrient uptake observed within one fungal species, as shown in the yeast experiments, emphasizes the importance of comparative study between two genetically distinct species.

Previously, we used LIBS to analyze nutrient elements from fresh samples of poplar roots and soil and to investigate the plant nutrient transport seen under different environmental conditions (27–29); therefore, we predict that the same procedure can be leveraged profitably with fungal biomass to provide a robust ionomic profile. We hypothesize that LIBS can be used as a technique to (i) identify ionomic profile of axenic fungal cultures and (ii) understand how fungi ionomics profiles differ when grown in nutrient-poor or nutrient-rich media. Despite the challenges of using LIBS for simultaneous quantification of multiple elements and low-level (<100 ppm) quantification, it can still be a valuable tool for high-throughput screening. Because our goal is to determine whether LIBS can be used to distinguish axenic fungal cultures, we examined two genetically distinct fungi, *Hyaloscypha finlandica* and *Mucor hiemalis*, grown in a nutrient-rich medium called potato dextrose broth (PDB) and a nutrient-poor medium called glucose minimal medium (GMM) broth after 21 days of growth at 25°C. Both fungi were selected because (i) they are commonly isolated as endophytes from *Populus trichocarpa* x *deltoides* genotype 52-225 (30), (ii) we can easily grow axenic cultures in liquid broth and solid agar media, (iii) they have different physiological structures and growth rates, and (iv) they would presumably be the first representatives of fungal ionomics profiles. Our study will provide baseline data set results from axenic isolates, specifically those not derived from yeasts or mushrooms collected from a market or nature, which can be colonized by other microbes or infiltrated with debris or soil particles. *H. finlandica* is an ascomycete and dark septate endophyte that has a mutualistic lifestyle with poplar trees. *M. hiemalis* is a ubiquitous mucoromycete with a saprotrophic and pathogenic lifestyle in soil and humans. After 21 days of growth at 25°C, we identified 15 elements through LIBS.

## MATERIALS AND METHODS

### Isolates used and preparation of fungal biomass for LIBS analysis

**Fig 1:**
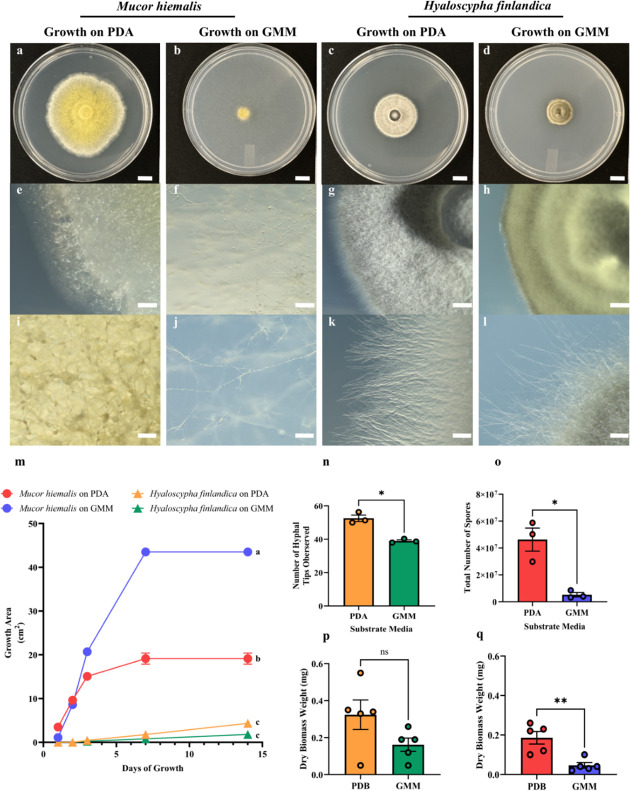
Growth and development of *M*. *hiemalis* and *H. finlandica* on potato dextrose agar (PDA) or broth (PDB) and glucose minimal medium (GMM) agar or broth. (a–d) Macroscopic photos display different physiologies and color appearances of the same fungi across different substrate media. Scale bar is 1 cm. Hyphal development on the edges (e–h), and scale bar is 1 mm. Closer image of hyphal development (i–l), and scale bar is 200 µm. (**m**) Growth rate of the fungi on different growth media, (**n**) development of hyphal tips in *H. finlandica*, (**o**) sporulation in *M. hiemalis,* (**p**) *H. finlandica* biomass, and (**q**) *M. hiemalis* biomass growth in each substrate that was used for the ionomic profiling. *M. hiemalis* did not produce distinguishable hyphal tips to be counted, and *H. finlandica* did not produce spores in our experiment. For growth rate, one-way ANOVA was significant (*P*-value = 0.0001), and Tukey's post-hoc analysis was used, where different letters indicated significant differences. For hyphal tips and sporulation counts, a Student's *t*-test was used, where (*) indicates *P-value <* 0.05; (**) indicates *P*-value < 0.01; and (ns) indicates not significant. There were five biological replications per condition. The error bars in all figures indicate the standard error of the mean.

*H. finlandica* (syn: *Cadophora finlandica*) strain PMI 746 and *M. hiemalis* strain PMI 3043 were used for this experiment. They were grown in sterile 125 mL Erlenmeyer screw cap flasks filled with 50 mL of PDB (BD Difco, New Jersey) or GMM without 1% thiamine (31) at 25°C in the dark at 250 rpm using an Eppendorf New Brunswick Excella E25 incubator shaker. There were five biological replications per condition. The fungi were grown for 21 days because *H. finlandica* grows slower compared to *M. hiemalis* in potato dextrose agar (PDA) and GMM agar (Fig. 1)*,* and the goal was to compare the ionomics profiles equally. After 21 days, fungal biomass was collected in a 50 mL sterile Falcon tube and centrifuged at 4,000 rpm for 10 minutes, and then the supernatant was removed. Then, 25 mL of sterile Millipore water was added to the fungal biomass, vortexed for 2 minutes, and centrifuged again, and the supernatant was then removed. This process was carried out twice. After washing the fungal biomass, samples were placed in liquid nitrogen and stored at −80°C. Samples were lyophilized until dried, weighed, and autoclaved on a dry cycle at 121°C, 15 psi for 30 minutes before being removed from the containers, sectioned, and mounted on double-sided carbon tape (Nisshin EM. CO., LTD) affixed to glass slides. Samples were autoclaved to prevent viable fungal spores or hyphal from contaminating the LIBS system. Carbon tape with mounted samples was re-covered with tape backing and compressed for 48 hours prior to analysis.

### Fungal physiology analysis

Both fungi were grown on PDA (BD Difco, New Jersey; cat. no. 247940) for 30 days at 25°C in the dark, prior to the experiment. Afterwards, a sterile core borer with a diameter of 0.5 cm^2^ was used to cut fungal plugs. The plugs were transferred to PDA or GMM with agar (Sigma-Aldrich; cat. no. 05040) to take physiology measurements. The growth rate was determined by measuring the diameter of the fungi 1-day post-inoculation (dpi), 2 dpi, 3 dpi, 7 dpi, and 14 dpi at 25°C in the dark. The plug's 0.5 cm^2^ diameter was excluded from the final growth area. Macroscopic and microscopic photos were taken with a Zeiss SteREO Discovery v12. There were three biological replications per fungus and substrate media. Sporangiospores were collected from *M. hiemalis* grown on PDA and GMM. Next, 5 mL of sterile Millipore water was placed in each plate, and a sterile L-shaped spreader was used to disrupt the sporangiospores. Then, the sporangiospores were collected in a sterile 15 mL Falcon tube and vortexed for 3 minutes at maximum speed. Ten microliters of spore suspension was collected and placed in the Countess Cell Counting Chamber Slide and analyzed by the Countess II FL Automated Cell Counter. We had two technical replications per biological replication. Spore counting was not performed for *H. finlandica* because spores were not produced in culture. Hyphal tips were counted and analyzed for *H. finlandica* using a ZEISS SteREO Discovery v12. Every visible hyphal tip within a frame of the image was counted. There were three technical replications per biological replication. Hyphal tips were not analyzed for *M. hiemalis* because no distinguishable tips were observed.

### LIBS analysis

Measurements were performed using a LIBS-8 module from Applied Photonics; this instrument utilizes a 1,064 nm Nd:YAG laser passed through beam expansion optics prior to focusing onto the sample to provide a tighter spot size of approximately 100 µm. A laser pulse energy of 100 mJ was used to provide strong signal-to-noise levels. Prior to testing samples, metal coupons were measured to ensure proper LIBS performance. Each sample was tested in a 4 × 4 grid with one shot per spot, providing 16 spectra per sample. The plasma light was collected using eight collimating optics located above the sample, off-axis from the impending laser pulse. The light was transported to an eight-channel spectrometer with a range of 178–1,022 nm. This spectrometer bank was operated with a delay time of 3 µs and an integration time of 50 µs.

The results of every four shots were averaged to provide four spectra per sample, with five technical replicates, leading to 20 spectra per biological specimen. The spectra were inspected to identify all elemental emission peaks, and the background-corrected peak intensities were tabulated for each spectrum. The elemental emissions were compared to each other to investigate the Pearson correlation coefficients, providing insight into the unique fungi sample profiles. A positive correlation coefficient indicates two variables having a positive linear correlation; conversely, a negative correlation coefficient indicates a negative linear correlation. The linear correlation between two variables is categorized as small, medium, or large if the magnitude (absolute value) of the correlation coefficient value is 0.1–0.3, 0.3–0.5, or 0.5–1.0, respectively. A magnitude value of <0.1 is categorized as having no linear correlation. Principal component analysis (PCA) was applied to the collected spectra, and the principal component (PC) loadings were compared to the elemental profiles to identify the behavior captured by each PC. Lastly, the PCA transformed data were modeled using a linear classification model to distinguish fungi samples using only LIBS elemental profiles.

### Statistical analysis

Fungal physiology statistical analyses were performed using the GraphPad Prism software version 10.2.0 (392) (GraphPad, San Diego, California). For fungal growth rates, one-way analysis of variance (ANOVA) was performed per time point and condition. A Tukey's multiple comparison test was performed per time point and condition. Similar letters indicate no significant differences, whereas different letters indicate significant differences (*P*-value < 0.05). For the number of hyphal or spores counted, a paired *t*-test was performed. For the dry biomass comparison, an unpaired *t*-test was performed within a species and between the two-substrate media. For both *t*-test analyses, asterisks indicate significant differences (*P*-value < 0.05). The error bars in all figures indicate the standard error of the mean.

## RESULTS AND DISCUSSION

First, we determined differences in growth rates, biomass weight, sporulation, number of hyphal branching tips, and appearances in the nutrient-rich and nutrient-poor media between the fungal species (Fig. 1).

**Fig 2:**
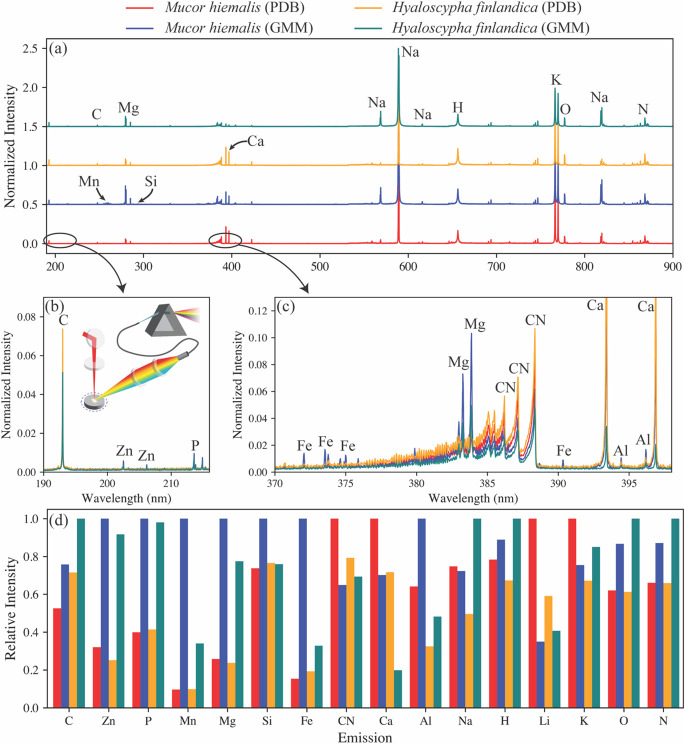
The averaged and normalized LIBS spectra for each fungi species in the different growth media are shown with (**a**) an offset to compare the labeled major emission peaks. Multiple peaks may be seen for a single element due to the branching electronic transitions as the plasma cools. A notional diagram of a LIBS measurement and a closer look at the ultraviolet carbon, zinc, and phosphorus emissions are shown in (**b**). A similar zoomed plot of the 370–400 nm region (**c**) shows the iron, magnesium, calcium, and aluminum elemental emissions and the carbon–nitrogen (CN) molecular bands. The average relative emission intensities are compared for each element in (**d**). Data are based on the average of five biological replications per condition.

Next, we compared the averaged and normalized LIBS spectra for the different sample groups (Fig. 2a). At the larger scale, the LIBS profiles appear nearly identical, but unique spectral fingerprints begin to emerge upon closer investigation (see Fig. 2b and c). The spectra were parsed from 190 nm to 900 nm to identify all element signatures within the sample set. The elements identified include carbon, zinc, phosphorous, manganese, magnesium, silicon, iron, calcium, aluminum, sodium, hydrogen, lithium, potassium, oxygen, and nitrogen.

The relative emission intensities of all these elements were compared to one another to investigate trends in the elemental profiles of the fungi species and to discern any effects of the growth media. Several trends were identified when comparing the fungi species. *H. finlandica* showed consistently more intense emissions from zinc, phosphorus, manganese, magnesium, iron, hydrogen, and oxygen, regardless of the growth media. The mutually elevated hydrogen and oxygen levels likely indicate that *H. finlandica* retains moisture to a greater extent than *M. hiemalis*, regardless of the growth media. *M. hiemalis* exhibited more intense lithium and calcium emissions. *M. hiemalis* showed lower aluminum levels than *H. finlandica* grown in the same media. Higher calcium and aluminum levels were detected in fungi grown in PDB, while specimens grown in GMM had more intense carbon emissions.

**Fig 3:**
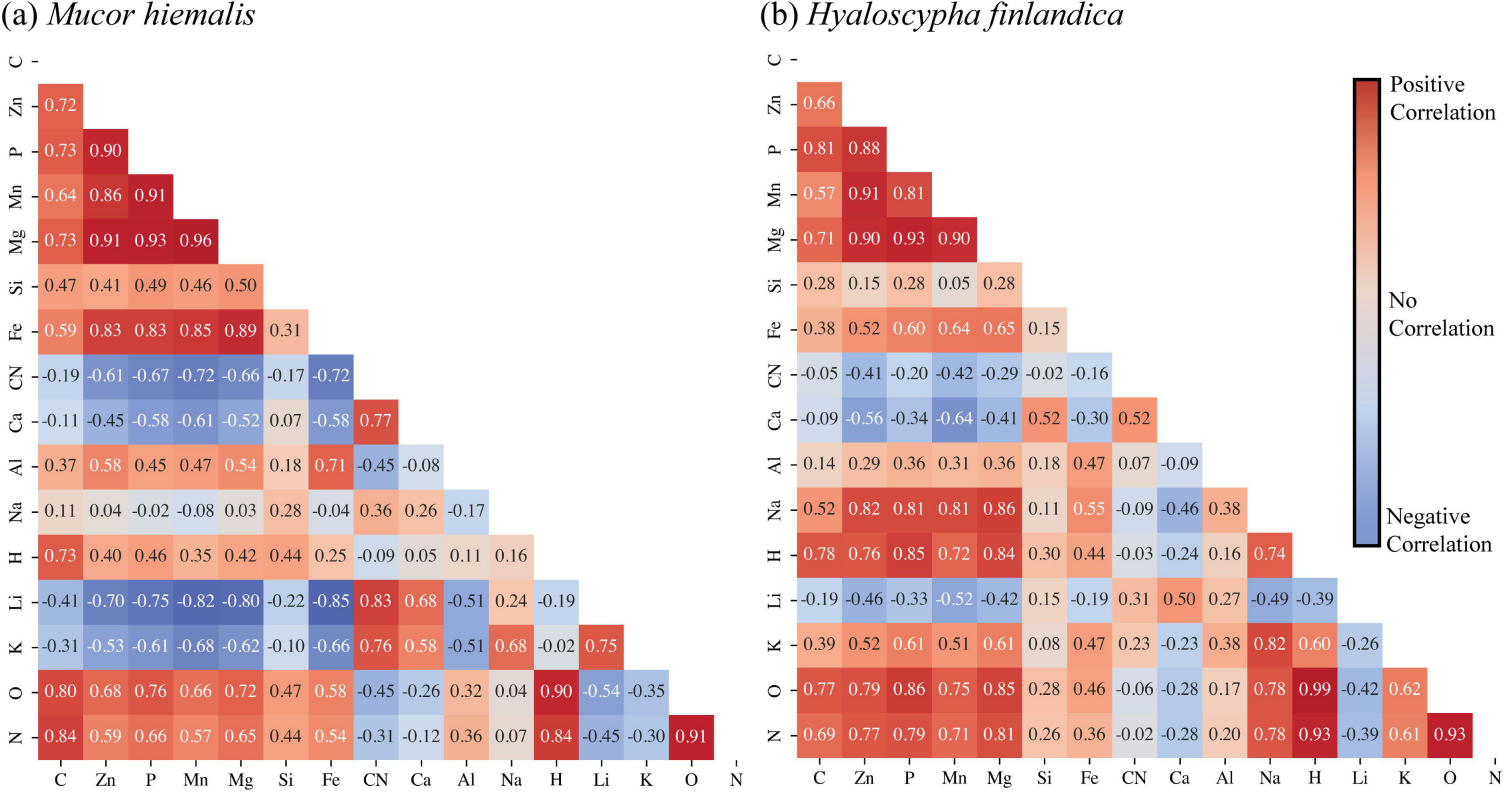
Pearson correlation coefficients between elemental emissions of (**a**) *M. hiemalis* and (**b**) *H. finlandica.* Each cell represents the Pearson correlation coefficient between the row and column labels. Positive correlations are shown in red, and negative correlations are shown in blue.

The emission signal intensities for each sample type were compared to one another through Pearson correlation analysis to investigate how elemental signatures related to one another. The Pearson correlation coefficients for *M. hiemalis* (Fig. 3a) and *H. finlandica* (Fig. 3b) are visualized in Fig. 3. For both *M. hiemalis* and *H. finlandica*, the correlation coefficients indicate strong positive correlations with carbon, zinc, phosphorus, manganese, and magnesium emissions. The positive correlations between these elements in both species may indicate their vital role in fungi propagation and survival. For simplicity, this group of elements (i.e., carbon, zinc, phosphorus, manganese, and magnesium) is referred to as *essential elements* for the remainder of the correlation analysis. Similar to the essential elements, hydrogen, nitrogen, and oxygen exhibit strong positive correlations regardless of the species, but this result is likely due to contributions from both sample moisture and the atmospheric contributions from these elements when LIBS is not performed under an inert cover gas. When the correlation coefficients of each fungi species are compared, a few noticeable differences are seen. Firstly, *H. finlandica* exhibits strong positive correlations among sodium, hydrogen, and the essential element group. This indicates a reliance of *H. finlandica* on sodium that *M. hiemalis* does not exhibit. Similar behavior was observed for potassium in *H. finlandica*, but generally, a medium positive correlation exists between the essential elements and potassium. *M. hiemalis* shows a robust negative correlation between potassium and essential elements. It shows stronger positive correlations among silicon, iron, and the vital elements; although *H. finlandica* shows a positive correlation between silicon and calcium, *M. hiemalis* does not. Both fungi species show negative correlations between lithium and the essential elements, although *M. hiemalis* shows a stronger negative relationship. This likely indicates that lithium harms fungal growth. The last significant difference between the two fungi species is *M. hiemalis*' strong positive correlation among aluminum with zinc, magnesium, and iron.

**Fig 4:**
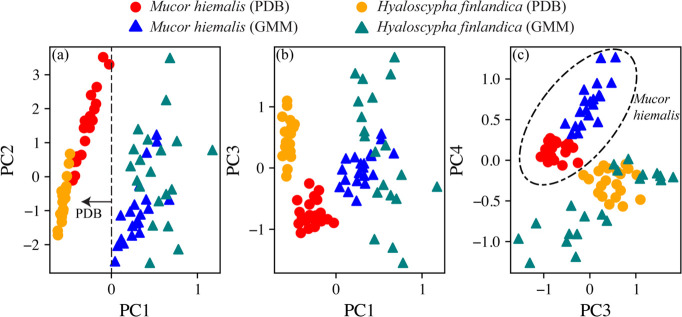
PCA score plots. Markers are colored by the sample class, and markers indicate the growth media; the PCA explained that variance ratios were determined to be as follows: PC1, 87.2%; PC2, 8.11%; PC3, 2.06%; PC4, 1.17%; and PC5, 0.57%.

In addition to correlation analysis on the emission intensities, PCA was applied to the entire LIBS spectrum to further investigate the key drivers for the sample set's variance. PCA score plots are shown in Fig. 4, with the markers colored by fungi species and growth media. Five PCs were used to explain a total of 99.11% of the signal variance in the data set. The score plots show that the samples form clusters around their fungi species and growth media—meaning PCA loadings can be used to decipher which signals are heavily impacted by these factors. PC1 clearly captures the variance related to the growth media used (Fig. 4a), where a negative PC1 score indicates PDB and a positive score points to GMM. Together, PC1 and PC3 begin to cluster the various fungi species, particularly *M. hiemalis* and *H. finlandica* grown in PDB, as seen in Fig. 4b. The score plot shown in Fig. 4c reveals that PC4 is a strong indicator of the fungal species.

**Fig 5:**
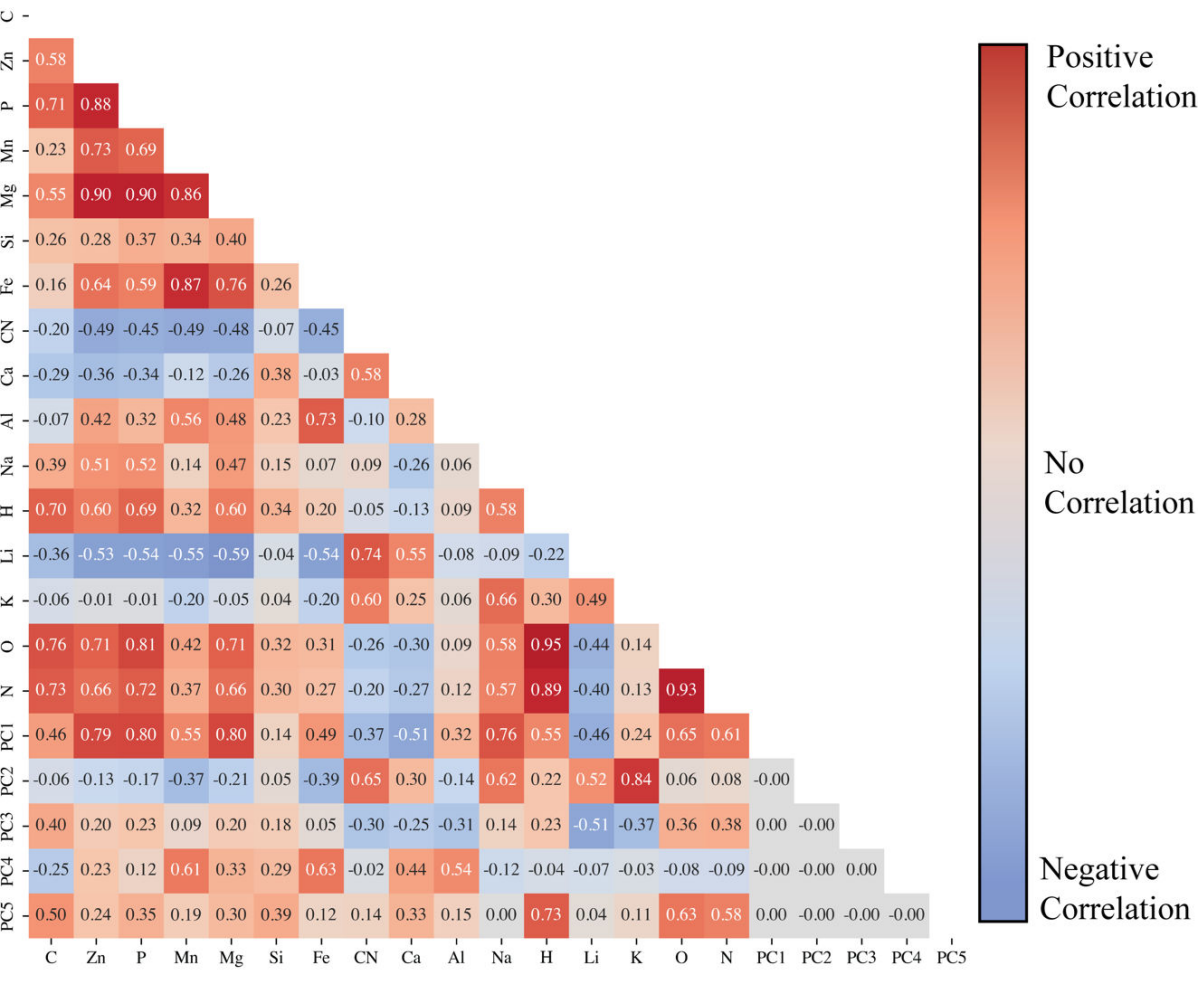
Pearson correlation coefficients between elemental emissions and PCs. Each cell represents the Pearson correlation coefficient between the row and column labels. Positive correlations are shown in red, and negative correlations are shown in blue.

To further investigate the individual PCs, the PCA scores for each sample can be added to the data matrix and correlated directly with the emission intensities. The Pearson correlation coefficient values relating the PCA scores to the emission intensities are tabulated in Fig. 5.

PC1 was observed to correlate strongly with sodium, hydrogen, oxygen, nitrogen, and essential elements. It shows a strong negative correlation with calcium and medium negative correlations with lithium and the CN molecular emission. Because a negative PC1 score can be used to discern when the samples are grown in PDB, we would expect more intense calcium, lithium, and CN emissions in the PDB samples. This behavior is verified in the spectra shown in Fig. 2d. PC4, which largely separates fungal species, shows strong positive correlations with manganese, iron, and aluminum and medium positive correlations with magnesium and calcium. Accordingly, *M. hiemalis* grown in GMM has the strongest manganese, iron, and aluminum emissions (see Fig. 2d), and *M. hiemalis* consistently has greater magnesium and calcium emissions than *H. finlandica* grown in the same media.

The PCA analysis allows for a more exhaustive interpretation of the elemental profiles of the samples tested. Both growth media and fungi species can be distinguished visually on score plots. Therefore, the final step was to test the ability to classify fungi samples based on LIBS elemental spectra. The sample spectra (*n* = 80) were randomly split into thirds: one-third was used to train the PCA classifier, and two-thirds were used to test the classifier. PCA classifier models were built to simultaneously predict growth media, fungi species, or both. The models predicted the test group classes with 100% accuracy for both the growth media and fungi species classifiers. The combined classifier predicted the test group classes with 98% accuracy; only one *M. hiemalis* grown in GMM was falsely predicted to be *H. finlandica* grown in PDB. If the average number of shots is increased from four to eight, then the classifier accuracy rises to 100%.

Linking the phenotype to ionomic profiles would indicate that the elemental fold-increase in aluminum, carbon, hydrogen, iron, manganese, magnesium, nitrogen, oxygen, phosphorus, and zinc accumulated in the *M. hiemalis* biomass when grown in GMM may have influenced its faster growth rate, transparent appearance, and reduced biomass and sporulation in comparison to PDA/PDB. Conversely, the fold-increase in calcium, lithium, and potassium in *M. hiemalis* biomass when grown in PDA or PDB could have been the cause for its slower growth rate, opaque yellow appearance, and increased biomass and sporulation when compared to GMM. For *H. finlandica,* differences in nutrient-rich or nutrient-poor media did not produce a significant difference between growth rate and biomass. However, when *H. finlandica* grew in PDB, a fold-increase of calcium and lithium could have impacted the increased hyphal tips, its uniform appearance, and the gray opaque appearance observed when grown on PDA. Interestingly, more increase-fold of elements like aluminum, carbon, hydrogen, iron, manganese, magnesium, nitrogen, oxygen, phosphorus, potassium, sodium, and zinc could have negatively influenced the growth rate and the number of hyphal tips and its sporadic formation. Moreover, these elements could have contributed to the yellow-green opaque appearance of the fungus.

### Conclusion

This study demonstrates that LIBS can be used successfully to generate rapid, high-quality elemental fingerprints for fungal biomass with minimal sample preparation. To our knowledge, this is the first study to examine endophytic fungi grown axenically in liquid broth media of various substrates and that were not yeasts or mushrooms collected from nature or purchased from a market. LIBS can detect fungal elemental composition to the parts per million (ppm) level with minimal sample preparation. On average, between 0.2 and 0.6 mg of fungal biomass was needed to obtain the data sets. We concluded that elemental profiles differ based on the nutrient-rich or nutrient-poor environments or based on the fungal taxonomic assignment for calcium, hydrogen, iron, sodium, and silicon. Despite the nutrient-rich or nutrient-poor environments and taxonomically different fungal species, both fungi will uptake carbon, zinc, phosphorus, manganese, and magnesium, indicating that these elements may be vital for their survival and propagation. The results of this screening exercise could be used to plan future studies targeted at the impacts of specific nutrients.

Although we believe that LIBS will be a tool of the future for high-throughput ionomic profiling of fungi, there are inherent limitations. The main limit is that when used as a high-throughput screening tool as in this study, LIBS is only qualitative. For full quantification, matrix matched standards would be needed, which would likely lead to fungal samples being measured using a standard addition (24). This would require more fungal biomass, limit throughput, and result in only a few elements quantified at a time. Fortunately, qualitative elemental profiles still provide powerful insight into fungal behavior in different environments and additional analytical techniques could be leveraged for further investigation after LIBS screening as required.

This successful demonstration that LIBS can provide biological context about the mechanisms by which a microbe may acquire elements within its biomass sets the stage for future investigations using LIBS as a high-throughput phenotypic tool to provide insights into the elements required for fungal survival and propagation. We are currently undertaking experiments to determine whether ionomic profiling is conserved between species within the same taxonomic class; whether there is a variation among profiles within isolates of the same species; whether fungi that are classified as mutualists, saprotrophs, or pathogens cluster together when growing on the same substrate media; and whether those classifications merge when they are grown on nutrient-poor media.

## Data Availability

Raw data for fungal physiology (Fig. 1) are found in Table S1 and compared elemental compositions (Fig. 2) are found in Table S2. Fungal strains are available upon request.

## SUPPLEMENTAL MATERIAL

Captions msystems.00919-24-s0001.docx Captions for supplemental tables.

Table S1 msystems.00919-24-s0002.xlsx Raw data for fungal physiology.

Table S2 msystems.00919-24-s0003.xlsx Raw data for compared elemental emissions.

ASM does not own the copyrights to Supplemental Material that may be linked to, or accessed through, an article. The authors have granted ASM a non-exclusive, world-wide license to publish the Supplemental Material files. Please contact the corresponding author directly for reuse.
