# Supplementary material for: Fungal elemental profiling unleashed through rapid laser-induced breakdown spectroscopy (LIBS)
Source: mSystems. 2024 Aug 27;9(9):e00919-24. doi: 10.1128/msystems.00919-24 (PMC11406887; doi:10.1128/msystems.00919-24)
Supplement: Captions — for supplemental tables. [file msystems.00919-24-s0001.docx]

**Legend for Supplementary Tables:**

Table S1: Raw data for the fungal physiology.

Table S2: Raw data for the compared elemental emissions.
